# “Sacbe”, a Comprehensive Intervention to Decrease Body Mass Index in Children with Adiposity: A Pilot Study

**DOI:** 10.3390/ijerph15092010

**Published:** 2018-09-14

**Authors:** Ana Rodriguez-Ventura, Arturo Parra-Solano, Daniel Illescas-Zárate, Minerva Hernández-Flores, Carolina Paredes, Carmen Flores-Cisneros, Bernarda Sánchez, Maricruz Tolentino, Reyna Sámano, Daniela Chinchilla

**Affiliations:** Instituto Nacional de Perinatología, Department of Nutrition and Bioprogramming, 11000 Mexico City, Mexico; arturoparrasolano@gmail.com (A.P.-S.); daniel_i_z@hotmail.com (D.I.-Z.); minerva.hernandez.flores@gmail.com (M.H.-F.); caro.90.paredes@gmail.com (C.P.); malinali2000@yahoo.com (C.F.-C.); emiberna20@yahoo.com.mx (B.S.); cruz_tolentino@yahoo.com.mx (M.T.); ssmr0119@yahoo.com.mx (R.S.); danielachinchilla87@gmail.com (D.C.)

**Keywords:** adiposity, obesity, overweight, comprehensive interventions, BMI z-score, healthy lifestyle, sociocultural context

## Abstract

Interventions in children with adiposity decrease less than 0.2 the body mass index (BMI) z-score less than 0.2 and only in 21–23% of cases. Experts recommend focusing on the habits of a healthy lifestyle (HLS) but considering the sociocultural context of children and their parents. Our objective was to achieve a higher percentage of success in lowering the BMI z-score in children with adiposity and their parents through a pilot program “Sacbe” based on HLS, sensitive to the sociocultural context previously explored and with the active participation of parents. This is a pilot study in children aged 8 to 18 years with adiposity according to the BMI z-score. The program consisted of two workshops on HLS and nutrition given by the pediatric endocrinologist in group sessions with 3–5 families and reinforcements in each visit by registered dietitians. We recorded lifestyle habits and anthropometric characteristics of children and their parents at the baseline visit and every month for 3–4 months. Forty-nine families, 55 children and 64 parents participated, 60% of the children were female, the average age was 13.95 ± 3.3 years, 72.7% and 86.7% lowered the z score of the BMI due to intention to treat and protocol analysis (*p* < 0.001), respectively; BMI z-score decreased by 0.22 ± 0.21, from 2.13 ± 0.57 to 1.91 ± 0.58 (*p* < 0.001). In total, 83% of the parents involved were mothers, the average age was 45.8 ± 9.4 years, 77% lost weight and body fat (*p* < 0.001), the frequency of unhealthy habits decreased. The results of “Sacbe” exceeded expectations by combining the active participation of parents, sessions in groups, and the education on various components of an HLS inside sociocultural context. The main challenge will be to standardize and reproduce this type of complex interventions, as well as to assure long-term success.

## 1. Introduction

The prevalence of overweight (Ow) and obesity (Ob) has increased in the last two decades in the USA and the world [1]; Ob is present in 15% and Ow in 16.5% [2,3] of children from 6–19 years old. The prevalence is higher in Latin and African American vs. Caucasian children [4]. In Mexico, 35% of adolescents and children are Ow or Ob [5]. 

In a metaanalysis [6] that included 64 controlled studies (5230 adolescents), with 12 focused on lifestyle, 6 on diet and 36 on behavior, there was a reduction of weight in 6–12 months with or without medications (orlistat or sibutramine) and reported only 21% of success in decreasing BMI. Germann JN et al. [7] reported that out of 55% of adolescents that finished their participation for 23 months, only 23% of them decreased BMI significantly. Several authors concluded that interventions in lifestyle and behavior decrease weight significantly, but it is necessary to consider sociocultural features, improving relations between physicians and families and to calculate cost-effectivity [8,9,10,11]. Educative interventions may change behavior only if we consider the psychosocial context, if barriers to change are resolved and if the methods are effective [12]; also Physicians, Nurses, Registered Dietitians (RD), Psychologists and Exercise Physiologists need to work as a real team to improve outcomes [13]. In addition to family participation [14,15,16], group sessions have shown efficacy to decrease weight [17].

Interventions should consider the poor understanding of Ow/Ob consequences, associated comorbidities and individual, family and social barriers [18,19], in fact, there is evidence that when people feel the imposition of change without freedom to decide, they do quite the opposite. For this reason, it is important that children and their parents understand why they need to make changes to improve their weight through education [20]. People with diabetes risk factors want to obtain all the information from their physicians in a very simple and clear way with visual material to improve their understanding [21]. Some interventions have used the cognitive-behavioral model [22,23,24] with modest results, but it is important to complement it with the precede-proceed, the sociocultural [25] and the ecological [26] models. 

The Diabetes Prevention Program (DPP) [27] and TODAY [28] (Treatment Options for type 2 Diabetes in Adolescents and Youth) studies used an educative program about a healthy lifestyle to prevent diabetes in adults and treat type 2 diabetes in adolescents, respectively. Therefore, we designed a clinical and nutritional education program (CNEP) that we decided to name “Sacbe” (Mayan word that means “the white way”) because Mayan culture represents a common historical root among some Latino people and initially, this program was made in order to offer an alternative to Latino children in Boston even without type 2 diabetes. It is a comprehensive program about a healthy lifestyle based on DPP and TODAY studies complemented with other important habits related to Ow/Ob and considered the sociocultural context of participants [29]. Our aim was to investigate if we achieve greater success by combining different strategies and considering the sociocultural context in a comprehensive program in order to improve habits and decrease BMI in children with adiposity and their parents.

## 2. Materials and Methods

### 2.1. Study Design

This is a pilot quasi-experimental study to improve lifestyle habits in order to decrease BMI. “Sacbe” is a clinical and nutritional education program (CNEP) to learn about the diabesity epidemic and scientific evidence about a healthy lifestyle in order to decrease BMI inside a sociocultural context previously analyzed [29]. Inclusion criteria were 8–18 years old, any gender and ≥1 standard deviation (SD) BMI z-score. Exclusion criteria were having any other chronic diseases (lupus, diabetes, cancer, cardiac or renal diseases, etc.), and/or taking any medication related to their weight (metformin, cortisone, etc.) or any limitating physical condition. Elimination criteria were missing two or more visits or taking any other medication related to weight outcomes. Using sample size to detect a significant difference between 2 proportions, with a confidence interval of 95% and a power of 80%, considering that the reported success rate to decrease BMI is 23% [7], 15 patients were needed, however, taking into account the high rates of abandonment to treatment, we increased the sample size to 55 study participants. Fifty-four percent of participants came from 2 schools, a secondary and a high school and the rest came from our own Institute in the Pediatrics Department. We tried to have a control group by randomization but it was not possible because all participants wanted to participate in the workshops.

### 2.2. Data Record

Medical record, lifestyle habits (including physical activity and sedentarism), diet (24-hour food recall and a frequency of food consumption questionnaire) and anthropometric data (weight, height, BMI and blood pressure, fat in kg and percentage) were registered. Parents were equally evaluated (medical record, lifestyle habits, diet and anthropometric variables). Blood pressure (BP) was taken by the same nurse each visit during a period of 1.5 h approximately 3 times, mean BP was calculated. The size of the cuff used corresponded with the circumference of the upper arm.

#### 2.2.1. Adiposity Measurements 

Each visit, RDs obtained direct height measurements to the nearest 0.1 cm using a stadiometer (seca, model 242) and body weight to the nearest 0.1 kg using a scale and body composition analyzer (Biospace, inbody 370/JMW140) for children and/or adolescents and their parents or tutors. Body composition measurements were obtained with Bioelectrical Impedance Analysis (BIA) with plantar and hand electrodes: the output data provides the fat-free mass (FFM), fat mass (FM) and percentage body fat (PBF) in order to monitor progress or change on subsequent visits. BMI for age z-scores were computed with the use of WHO software AnthroPlus in order to classify youth as Ow/Ob. BMI z-scores provide an indirect age and gender specific measure of relative adiposity. 

#### 2.2.2. Assessment of Meal Patterns and Lifestyle 

Meal patterns and lifestyle were evaluated through a questionnaire administered by trained and standardized interviewers. The interview also included a 24-hour food recall [30,31]. Children and adolescents self-reported their data with the support of their parents or guardians when needed. According to the literature [32,33,34,35,36,37,38], the unhealthy habits that we considered were: irregular breakfast, eating very fast (less than 20 min), sleeping less or more than recommended by age group according to the National Sleep Foundation recommended sleep hours [39], eating out, watching television for more than 2 h, doing exercise for less than 2.5 h per week, sitting for more than 8 h and eat less than one serving of vegetables or fruits per day. All the patients received a pedometer and feeding plans designed by an RD according to their estimated energy needs and healthy habits were reinforced in the workshops and in each consultation by a Pediatric Endocrinologist and RD. Irregular breakfast was considered when breakfast is not eaten in the first 2 h upon awakening, which is associated with increased accumulation of fat. Eating in less than 20 min is associated with a lack of signaling of satiety to the brain. Eating out is associated with higher caloric intake and poor quality of food. Watching TV for more than 2 h is associated with adiposity due to the sedentary time and marketing exposition to improve buying hypercaloric products. Exercise for less than 2.5 h per week was the cut-off point to consider sedentarism because in adults this time is the minimum necessary to maintain weight. Eating less than 1 serving of fruit or vegetables a day was considered because our participants did not eat 5 serving of fruit or vegetables per day 

#### 2.2.3. Energy Intake

Energy intake was assessed at each clinical visit using face-to-face 24-h dietary recalls completed by RDs based on the multiple-pass procedure [31], which in a first step recorded an overview of all foods and beverages consumed during the previous day, then inquired details about amounts and type of each food and beverage, and lastly reviewed and verified the information and asked about food and beverages that are often forgotten. Total energy intake (kcal) was estimated using food composition tables comprising nutrient compositions of Mexican food (Sistema Mexicano de Alimentos Equivalentes) [40]. 

### 2.3. Intervention Description

In Sacbe, the CNEP was focused on a healthy lifestyle, following recommendations of DPP [27] and TODAY [28] studies, but additionally, we included the sociocultural and precede-proceed models proposed by Huang [25] to prevent diabetes as well as group sessions and family active participation. Sacbe consists of two workshops initially, one of them Medical and the other Nutritional, considering the sociocultural context previously evaluated [29]. Registered Dietitian (RD) visits were each month for 3–4 months to support diet knowledge and habits of a healthy lifestyle [41]. 

The Pediatric Endocrinologist reinforced healthy habits, explained workshops, diagnosis of nutritional status and risk factors. Visits with the Psychologists were necessary in some cases. These two workshops were held in groups of 3–5 families and they included the following topics:

- *Medical workshop*:
  1. Epidemic Ow/Ob and diabetes, consequences and risk factors. People wanted to get a clear explanation of the problem in order to change [18,19,20,21].
  2. Clinical evidence of benefits for participants in Prevention Programs focused on healthy lifestyle including type and quantity of food, exercise, sleep, meal schedule and positive attitude. People were interested in understanding the impact on their health related to these variables.
  3. Explanation about cutoffs of BMI in parents and children using CDC graphs in order to show their real weight status. Its importance relies on the lack of awareness about nutritional status and that Ow/Ob was initially not seen as a chronic disease [29,42].
  4. Strategies to improve their lifestyle habits organizing schedules, working as a family and empowering their individual decisions.
- *Nutritional workshop*:
  1. Food groups using the Mexican model “Plate of Good Eating” (Plato del Bien Comer [43]).
  2. Portion sizes of each food group using replica models.
  3. Healthy and unhealthy combinations of food groups.
  4. Examples of healthy menus and meal plan explanation.

### 2.4. Statistical Analysis

Statistical analysis was performed by SPSS version 22. We analyzed the distribution of variables and then we used parametric and non-parametric tests. In the bivariated analysis, we used Mc Nemar (dichotomic variables) and paired student´s *t* test or the Wilcoxon rank-sum test (continuous variables, according to normal vs. free distribution, respectively).

### 2.5. Ethical and Biosecurity Considerations

The Research and Ethic Committee of Mexico Children’s Hospital (Hospital Infantil de México Federico Gómez) and National Institute of Perinatology (Instituto Nacional de Perinatología) accepted this study. We obtained Informed Consent Letters signed from parents or tutors and children.

## 3. Results

Forty nine families, 55 children and 64 parents were accepted to participate in the program; they attended a baseline visit and completed 3 follow up visits, 9 children (16.7%) and 20 adults (31.3%) dropped out. Sixty percent were female, anthropometric data are reported in Table 1. According to the BMI z-score classification, in the baseline visit, obesity in children was present in 55.6% and it decreased to 48.9%; 58.7% of children lost weight, 72.7% and 86.7% lowered their BMI z-scores by intention to treat and per protocol analysis. The baseline BMI z-score was 2.13 ± 0.57 and in the last visit was 1.91 ± 0.58 (*p* < 0.001). In general, the frequency of unhealthy habits decreased their frequency, but only some of them were statistically significant (Table 2). Their median baseline caloric intake was 1557 (IQR 1210-1989) and in the third visit the median was 1501 (IQR 1138-1739) being statistically significant (*p* < 0.009). 

Eighty-three percent of parents were female, the mean age was 44.5 ± 9.4 years, mean height was 156.4 ± 9 cms, 90% presented Ow or Ob, 38 and 52%, respectively, and the global percentage decreased to 80% (41 and 39%, respectively) in the last visit. Seventy-seven percent lost weight and fat significantly and 50% lost ≥ 2.5% of their baseline weight (Table 3). Parents also decreased the frequency of unhealthy habits, but only one of them was statistically significant (Table 4); caloric intake did not change but the quality of food improved, they consumed 1320 (IQR 1115,1756) and 1445 (IQR 1199, 2389) calories in the baseline and last visits, respectively. 

## 4. Discussion

This comprehensive Clinical and Nutritional Education Program (CNEP) in families, “Sacbe”, achieved promising results to decrease BMI z-score and improve unhealthy habits in children and parents. In other studies, authors only reached a success rate of 21–23% with respect to decreasing the BMI of participants [6,7] and in this study, we achieved more than 72% of success. On the other hand, the decrease of the BMI z-score is usually less than 0.2 SD and only for a low percentage. We achieved a greater decrease in BMI compared to a well-conducted study by Rijks et al. [44] in which their participants with morbid obesity lost 0.13 SD throughout 1 year of follow-up while our participants lost 0.22 SD. It was also important to achieve our objective in a high percentage of participants despite limited resources and in a short period with few sessions. “Sacbe” could have achieved this promising result because this CNEP combines different strategies (group sessions, healthy lifestyle, family participation, interdisciplinary team) [11,12,13,14,15,16,17,28] that separately have worked and adds the active participation of parents (being equally evaluated compared with their children), as well as education about a healthy lifestyle and diabesity pandemic considering the sociocultural context of the participants [25,29].

Through recall 24 h recall and food frequency questionnaires, in addition to medical and nutritionist visits we observed that our participants (children and parents) improved the quality of their diet including more vegetables and decreased unhealthy food intake. They learned to identify the real status of their weight and recognize Ob/Ow as a chronic disease. In fact, recently, experts have recommended that Ob/Ow should be renamed ABCD, Adiposity-Based Chronic Disease [1] because people in general and even health personnel are not aware that Ow/Ob are chronic diseases and therefore they do not show any interest to get education about a healthy lifestyle or are not worried about improving their habits [29,30,31,32,33,34,35,36,37,38,39,40,41,42,43,44].

We achieved a significant reduction in BMI and fat in children and their parents in a brief period but the challenge will be to preserve this reduction long term. In Mexico City, in another study in preschoolers, with more human and financial support, the adherence to the intervention was very low and in those who remained for 6 months, there were no significant changes in their weight or BMI, only in their habits [45], however, experts recommend to first obtain changes in the habits of children [6,46,47]. In children and parents there was a decrease in unhealthy habits associated with adiposity, although, for children it was easier to change more habits and keep them stable for 3–4 months of follow-up than what it was observed in parents, as well as the adherence in terms of attendance to appointments. This could be because children have had unhealthy habits for less time and have fewer social responsibilities, in addition, there is a tendency to worsen habits over time [48,49]. On the other hand, psychological aspects should be evaluated because there is evidence that stress or anxiety can block the beneficial effects of a healthy diet [50]. In our study, participants reported less stress and anxiety facing this disease through group sessions and in family visits.

The side effects of this intervention included a tendency to eat less, especially in children by eliminating or decreasing the intake of processed products. A few patients reported family conflicts when they wanted to improve the quality of their food because not all family members were convinced to improve it.

The strengths of our study are the sample size that exceeded the one calculated to reach the main objective (percentage of children that reduce the BMI z score), the integration of the Sacbe work team, the acceptance of the parents (mainly mothers) to participate actively and the use of educational techniques within a sociocultural context previously analyzed [30]. Rajjo T. et al. [51] reported that comprehensive multicomponent interventions appear to have the best overall outcomes. Recently, 2 studies published [52,53] about long-term interventions long term in children showed that it is better to begin the first months of life of children and to consider the economic level because it may have a large difference in relation to outcomes if the population is at a low economic level. 

The areas of opportunity will be having a control group, increasing the sample size in order to analyze the role of each habit, quality of food, psychological evaluations, economic and education levels, and the cost effectiveness of this type of interventions. In order to prevent desertion it will be important to obtain permits from the work of the parents and the schools that the children attend, as well to provide economic compensation for transportation.

## 5. Conclusions

This program “Sacbe” decreased BMI in children and their parents at a high percentage, but the causal relationship cannot be established because of the quasi-experimental study design. The active participation of parents being evaluated in addition their children, group sessions and the education of a healthy lifestyle within their sociocultural context may explain the magnitude of these results. The biggest challenge is to confirm the effectiveness of this program and find strategies to ensure long-term success.

## Figures and Tables

**Table 1:** Anthropometric characteristics in children in baseline and 3rd. visits.

	Baseline Visit (*n* = 55) Median (IQR) Mean ± SD	Visit 3 (*n* = 44) Median (IQR) Mean ± SD	*p*
**Age (years)**	13.5 (11.4, 16.3)	14.2 (11.7, 17.9)	<0.001 †
**Weight (kg)**	63.5 (53.4, 77.0)	61.5 (52.1, 76.9)	0.177 †
**BMI z-score (SD)**	2.13 ± 0.57	1.91 ± 0.58	<0.001 *
**Fat (kg)**	26.7 (20.1, 31.4)	24.7 (16.4, 29.6)	0.207 †
**Fat (%)**	38.8 (34.3, 42.7)	37.3 (30.0, 42.3)	0.007 †
**Muscle (kg)**	22.7 (18.4, 25.8)	21.1 (17.5, 25.0)	0.518 †
**Systolic blood P (mmhg)**	106 (98, 116)	106 (100, 119)	0.806 †
**Dyastolic blood P (mmhg)**	71 (67, 79)	72 (66, 76)	0.916 †

† Wilcoxon, * paired Student´s *t*-test.

**Table 2:** Unhealthy habits of children in the baseline and 3rd. visits.

Unhealthy Habits	Baseline Visit N = 55 (%)	Visit 3 N = 44 (%)	*p*
Irregular breakfast	29 (53.7)	12 (27.3)	0.012 ‡
Eating < 20 min	12 (22.2)	6 (13.6)	0.549 ‡
Sleep time </> recommended by age on week	33 (61.1)	27 (61.4)	1.000 ‡
Sleep time </> recommended by age on weekend	22 (40.7)	12 (27.9)	0.210 ‡
Eating out	32 (59.3)	13 (29.5)	0.774 ‡
Watch TV > 2 h/day	38 (69)	17 (38.6)	<0.001 ‡
Exercise < 2.5 h/day	27 (49)	18 (40.9)	0.238 ‡
Eating < 1 serving vegetable/fruit/day	42 (76.3)	21 (47.7)	<0.001 ‡

‡ McNemar.

**Table 3:** Anthropometric data of parents in baseline and last visits.

	Baseline Visit (*n* = 64) Median (IQR) Mean ± SD	Last Visit (*n* = 50) Median (IQR) Mean ± SD	*p*
**Weight (kg)**	71.3 (64.4, 86.6)	69.2 (62.7, 78.7)	<0.001 †
**BMI (kg/m²)**	30.1 (27.2, 34.6)	28.8 (26.2, 33.1)	<0.001 †
**Waist (cm)**	95.0 (89.3, 103.0)	90.1 (83.5, 99.0)	<0.001†
**Fat (kg)**	27.7 (23.9, 35.3)	25.9 (21.6, 34.9)	0.009 †
**Fat (%)**	39.4 ± 7.4	38.0 ± 7.7	0.008 *
**Muscle (kg)**	24.3 (21.4, 28.0)	23.5 (21.2, 27.2)	0.406 †

† Wilcoxon, * paired student t-test.

**Table 4:** Unhealthy habits of parents in baseline and last visits.

Unhealthy Habits	Baseline Visit N = 64 (%)	Last Visit N = 50 (%)	*p*
Irregular breakfast	25 (39)	10 (20)	0.013 ‡
Eating < 20 min	11 (17.0)	7 (14)	1.000 ‡
Sleep < 6 h	10 (47)	8 (16)	1.000 ‡
Sleep ≥ 10 h on weekends	10 (15.6)	4 (8.3)	0.344 ‡
Eating out	33 (51.5)	15 (30)	0.077 ‡
Watch TV > 2 h/day	32 (50.0)	18 (36)	0.424 ‡
Exercise < 2.5 h/day	47 (73.4)	30 (60)	0.648 ‡
Eat < 1 serving fruit/vegetable/day	46 (72)	17 (34)	0.000 ‡

‡ McNemar.
